# The Influence of Sleep Disorders on Neurobiological Structures and Cognitive Processes in Pediatric Population with ASD and Epilepsy: A Systematic Review

**DOI:** 10.3390/ejihpe13110166

**Published:** 2023-10-27

**Authors:** Miguel López-Zamora, Alejandro Cano-Villagrasa, Antonio Cortés-Ramos, Nadia Porcar-Gozalbo

**Affiliations:** 1 Department of Developmental and Educational Psychology, University of Malaga, 29010 Malaga, Spain; miglopzam@uma.es; 2 Faculty of Health Sciences, Universidad Internacional de Valencia, 46002 Valencia, Spain; nadia.porcar@professor.universidadviu.com; 3 Health Sciences PhD Program, Universidad Católica de Murcia UCAM, Campus de los Jerónimos n°135, Guadalupe, 30107 Murcia, Spain; 4 Department of Developmental and Educational Psychology, Faculty of Educational and Sports Sciences of Melilla, University of Granada, 18071 Granada, Spain; antoniocortes@ugr.es

**Keywords:** childhood, ASD, epilepsy, sleep disorders, systematic review

## Abstract

Autism Spectrum Disorder (ASD) and epilepsy are increasingly prevalent comorbidities in our society. These two disorders are often accompanied by other comorbidities, such as sleep disorders, significantly impacting the quality of life of individuals with ASD and epilepsy. To date, clinical approaches have primarily been descriptive in nature. Therefore, this study aimed to analyze the relationship between ASD, epilepsy, and sleep disorders, exploring neurobiological dysfunctions and cognitive alterations. A total of 22 scientific articles were selected using a systematic literature review following the criteria established using the PRISMA model. The selected articles were gathered from major databases: Medline, PubMed, PsycINFO, Google Scholar, and Web of Science. Inclusion criteria specified that study participants had an official diagnosis of ASD, the article precisely described the evaluation parameters used in the study participants, and individual characteristics of the sleep disorders of the study participants were specified. The results indicate, firstly, that the primary cause of sleep disorders in this population is directly linked to abnormal serotonin behaviors. Secondly, significant alterations in memory, attention, and hyperactivity were observed. In conclusion, sleep disorders negatively impact the quality of life and neurocognitive development of the pediatric population with ASD and epilepsy.

## 1. Introduction

Autism Spectrum Disorder (ASD) is a neurodevelopmental disorder that typically begins in the first 30 months of life and affects the normal development of the brain in terms of social and communication skills [1]. Common characteristics of ASD encompass a wide and heterogeneous range of symptoms, including impaired social relationships [2], difficulties in verbal and non-verbal communication [3], problems processing sensory information [4], as well as restricted and repetitive patterns of behavior [5].

Between 5–40% of children with ASD develop epilepsy during their developmental course [6]. This high incidence appears to follow a bimodal distribution, with an initial peak between 1–5 years of age and a second peak during prepuberty and adolescence [7]. There is a significant difference in the rates of epilepsy in children with ASD without comorbid conditions compared to those who exhibit neurological disorders, such as intellectual disability, hypotonic syndrome, or cerebral palsy, which exhibit epilepsy. In this second group, epilepsy rates are higher, affecting more than 60% of patients [8].

Among the comorbid disorders in the pediatric population with ASD, sleep disorders (SD) stand out, primarily manifesting as difficulties in sleep onset, maintenance, nighttime awakenings, and sleep apnea [9]. Generally, individuals with ASD have a multifactorial etiology, tend to be chronic, and have negative consequences on the patient and/or the family environment, both in their progression and quality of life [10]. Sleep disorders (SD) are reported in 6–25% of the pediatric population, but this figure rises to between 50–95% in children with ASD and epilepsy [11]. SD in the population with ASD and epilepsy can be similar to those seen in the general pediatric population, with the most prevalent being onset insomnia, nighttime awakenings, and reduced total sleep time [12].

SD should hold a prominent place in the diagnostic and therapeutic stage for individuals with ASD and epilepsy. The pediatric population with ASD and epilepsy has proven effects on cognition and behavior, which become even more evident in patients with this comorbidity. These effects can be specific to certain genetic conditions and may, therefore, require a specific clinical approach and intervention [13]. The pathophysiology of SD in children with ASD and epilepsy is multifactorial, with some patients having a direct relationship with their specific genetic diagnosis or phenotype. SD in this population is often associated with common causes of sleep problems in the general pediatric population [14]. These factors are related to neurobiochemistry, seizures and epileptic syndromes, and specific cognitive impairments seen in this population. Regarding the neurobiochemistry of SD, various neurobiological anomalies have been described in ASD and epilepsy, highlighting alterations in neurotransmitters and hormones involved in sleep regulation [15]. On one hand, serotonin, through its modulation of cholinergic neurons in the brainstem, plays a critical role in suppressing REM sleep and promoting wakefulness [16]. However, a decrease in serotonin synthesis has been observed in children with ASD compared to those who do not have it [17]. On the other hand, melatonin, produced by the pineal gland, is a key regulator of circadian rhythms, and a decrease in melatonin during sleep has been reported in children [18] and young adults [19] with ASD and epilepsy. Therefore, due to the neurobiological alterations in children with ASD, sleep can be affected by this disorder. Additionally, it is observed that individuals with ASD who have epilepsy have a direct relationship with sleep disorders.

Epileptic syndromes, particularly those affecting the frontal lobe, are characterized by seizures that occur exclusively or predominantly during non-rapid eye movement sleep (NREM) [20]. Interictal epileptiform discharges (IEDs) are sharp waves or spikes that occur on an electroencephalogram (EEG) between seizures. IEDs refer to abnormal electrical brain activity between seizures in individuals with epilepsy. Chronic sleep deprivation can lower the seizure threshold, making it more likely for IEDs to trigger actual seizures. This connection underscores the importance of maintaining good sleep habits and managing sleep disorders in epilepsy patients to reduce the risk of seizures. Like seizures, IEDs are activated during NREM sleep and suppressed during REM sleep [21]. Chronic sleep deprivation can be caused by sleep disorders that disrupt and interfere with sleep, such as obstructive sleep apnea (OSA). In OSA, breathing pauses awaken the patient from sleep due to a protective response, resulting in fragmented sleep. A study on the treatment of OSA in children with ASD and epilepsy has shown an improvement in seizure control [22]. Other sleep disorders, such as insomnia, can also lead to sleep deprivation and worsen seizure control [23]. Therefore, paying attention to sleep in children with ASD and seizures and implementing appropriate treatment strategies can result in better seizure control.

Epileptic seizures themselves can impact sleep, and this influence is not limited to the moment of seizure onset. Complex partial seizures of the temporal lobe decrease REM sleep, especially when they occur during sleep but also when they happen the day before sleep onset [24]. These changes may result from disruption in circadian rhythms or hormonal influences resulting from seizures.

Regarding the cognitive impairments that seizures related to sleep and IEDs produce in learning, memory, and daytime behavior, these are still largely unknown and represent a rapidly expanding area of research [25]. Sleep-related problems have been shown to disrupt daytime behavior and academic performance [26]. Sleep-related problems are significantly more common in children with ASD and epilepsy. Researchers have attempted to establish a correlation between behavioral problems identified in epilepsy patients and various sleep indices. In Zwaigenbaum et al.’s study [27], it was found that rapid eye movement latency, apnea duration, and periodic leg movements in children with epilepsy correlated with depression, inattention, hyperactivity, and/or oppositional defiant disorder. Therefore, sleep disorders in the pediatric population with ASD and epilepsy constitute a comorbidity that negatively influences overall developmental progress, impairing the acquisition of skills such as communication, attention, emotional control, and the quality of life of this group.

Based on this, the aim of this systematic literature review was to compile relevant data from research on the relationship between sleep disorders and the pediatric population with ASD and epilepsy. Furthermore, the specific objectives were to analyze the neurobiological alterations related to sleep disorders and describe the cognitive performance related to the pediatric population diagnosed with ASD and epilepsy. The hypotheses of this systematic review were as follows: (I) It is expected that the neurobiological and neurochemical alterations described in children with ASD and epilepsy will disrupt brain activity and give rise to various sleep disorders, and (II) it is expected that sleep disorders in this population will limit cognitive competencies and alterations in basic and higher-order cognitive processes will be observed.

## 2. Materials and Methods

This work presents a systematic literature review on ASD and epilepsy and how sleep disorders are affected in this population. The question to be addressed is the relationship between sleep disorders, cognitive impairments, and emotional disturbances in family members in the pediatric population with ASD and epilepsy, following the criteria of the following PICO (Patient, Intervention, Comparison, Outcomes) model: (P) Individuals diagnosed with ASD and epilepsy. (I) Not applicable. (C) Not applicable. (O) Sleep disorders. To ensure the precision of the research question, criteria related to individuals with ASD and epilepsy were specified, comparing their symptoms and the results of their respective diagnostic assessments [28]. Furthermore, this literature review adhered to the guidelines and recommendations established in the Preferred Reporting Items for Systematic Reviews and Meta-Analyses (PRISMA) statement [29].

### 2.1. Search Strategy

An electronic search was conducted between March 2023 and July 2023 in the following databases: Medline, PubMed, PsycINFO, Web of Science, and Google Scholar. The search was limited to peer-reviewed articles published in journals and/or available online and written in Spanish or English. To ensure that the included articles were current, the search was limited to the last 20 years. The following terms in the article title, abstract, and keywords were considered in the study’s search: epilepsy, ASD, and sleep disorders.

Regarding the search strategy, the following terms were used, combined with Boolean operators: “(epilepsy) AND (ASD) AND (sleep disorders)”, “(epilepsy comorbidity) AND (ASD) AND (cognitive impairments)”, “(causes of sleep disorders) AND (ASD and epilepsy)”.

### 2.2. Study Selection Process

To obtain highly relevant published studies based on the study’s objectives and PRISMA guidelines [30], inclusion and exclusion criteria were established before the systematic review. To improve the search’s quality, a peer review was conducted using the standardized Peer Review of Electronic Search Strategies (PRESS) tool [31]. In this way, in the initial stage of study pre-selection, based on the information provided in the title and abstract, the following inclusion criteria were considered for the selection of relevant articles: (I) study samples reflect the comorbidities of the two conditions under study (epilepsy-ASD and sleep disorders); (II) these conditions have been specifically evaluated; and (III) evaluations have been conducted by professionals in psychology or medicine. As for exclusion criteria, the following were considered: (I) individual case reports without a methodological design; (II) studies of epilepsy unrelated to ASD or vice versa; and (III) reviews, editorials, or abstracts of studies presented at conferences.

In the second stage of study selection, a comprehensive analysis of articles meeting the specified inclusion criteria was carried out. In this process, the following inclusion criteria were considered: (I) study participants had an official diagnosis of ASD; (II) the article accurately described the parameters of the evaluation conducted on the participants; and (III) individual characteristics of sleep disorders in study participants were specified.

The process of selecting studies included in this systematic review was conducted by two researchers and consisted of four phases. In the first phase, scientific literature in the fields of psychology and medicine was reviewed in major databases. In the second phase, each researcher reviewed the title and abstract of each eligible article in accordance with the pre-established inclusion and exclusion criteria. In the third phase, articles meeting the inclusion and exclusion criteria underwent a detailed analysis using a thorough reading of the full text. Finally, in the fourth phase, the examination and exclusion of articles that did not address the topic of interest in this systematic review were completed, evaluating the main information from the selected articles.

### 2.3. Final Study Selection

**Figure 1:**
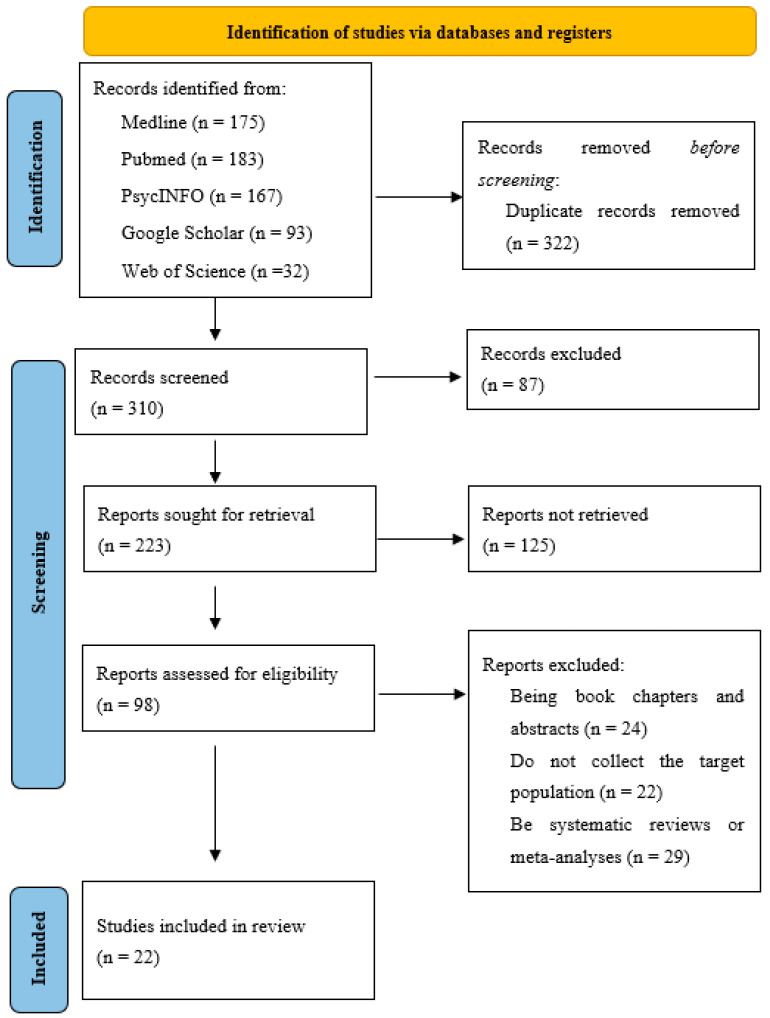
Modified PRISMA Diagram with the Studies Included in the Systematic Review.

During the initial search for studies, a total of 632 potentially eligible articles were found, distributed as follows: 175 from Medline, 183 from PubMed, 167 from PsycINFO, 93 from Google Scholar (via manual bibliographic search), and 32 from Web of Science. Figure 1 presents a flowchart illustrating the study selection process, following the PRISMA statement [32]. Out of this total, 322 duplicate references were discarded. Finally, a total of 22 articles have been selected and included in this systematic review (Table 1).

Lastly, regarding the methodological design of the selected studies, quasi-experimental studies with assessments at one or multiple points in time, both cross-sectional and intra-group and inter-group longitudinal assessments to evaluate differences, were conducted.

## 3. Results

The selection of the reviewed articles below follows the previously established inclusion and exclusion criteria, as well as being the most recent articles with the highest relevance and participant samples published to date. These are reported in thematic blocks in accordance with the proposed objectives.

### 3.1. Neurobiological Alterations of Sleep Disorders in the Pediatric Population with ASD and Epilepsy

Neurobiological alterations related to ASD and epilepsy lead to a dysregulation of the sleep-wake cycle in individuals with this comorbidity, primarily manifested via altered functioning of the neurotransmitters gamma-aminobutyric acid (GABA) and serotonin, as well as the neurohormone melatonin [33,34].

GABA, an inhibitory neurotransmitter of the central nervous system, acts as a sleep inducer by inhibiting the activity of neurons in the brainstem that are active during wakefulness [33]. Children with ASD and epilepsy exhibit dysregulation in GABA interneurons, which can be explained by the presence of mutations in chromosome 15q in some patients, which is related to the storage of GABA-related genes [34]. The identification of a susceptibility region for ASD and epilepsy with genes related to GABA suggests that changes in gene expression in this area could disrupt GABA’s inhibitory function, leading to hyperactivity and, consequently, insomnia in individuals.

The other neurotransmitter, serotonin, also plays a significant role in sleep cycles, as it promotes wakefulness and inhibits REM sleep, making it a key factor in studying sleep disorders in this population. Hyperserotonemia is present in more than 25% of children with this comorbidity and in many of their first-degree relatives. Research on the pediatric population with ASD and epilepsy has also suggested genetic variations related to serotonin transport and degradation [35]. Therefore, abnormal serotoninergic activity will impair sleep due to its impact on melatonin [36]. It has been proposed that elevated levels of serotonin in blood could be used as the first biomarker to identify patients with ASD and epilepsy [37].

Finally, melatonin, a hormone produced by the pineal gland, plays a critical role in regulating sleep-wake rhythms [38]. Its release is suppressed in the presence of bright light and increases in response to darkness [39]. Melatonin is synthesized from serotonin using the enzyme acetylserotonin O-methyltransferase (ASMT). Despite this population having low levels of melatonin and high levels of serotonin, research attention is focused on the gene that controls ASMT production [40]. Mutations or polymorphisms in this gene are associated with low melatonin levels in individuals with ASD and epilepsy, as well as certain autism-related traits like language impairments or social behavior in the general population [41,42]. Genetic variation in ASMT and another enzyme in the melatonin pathway, cytochrome P450 1A2 (CYP1A2), were identified in children with ASD and epilepsy and comorbid sleep onset delay [43,44]. Therefore, differences in sleep architecture based on genetic abnormalities provide further evidence of the neurobiological basis of sleep disorders in individuals with ASD and epilepsy.

### 3.2. Cognitive Impairments Caused by Sleep Problems in Individuals with ASD and Epilepsy

In the pediatric population diagnosed with ASD and epilepsy, it is crucial to analyze the relationship between sleep disorders and cognitive impairments. Sleep problems can exacerbate neurocognitive dysfunction in areas such as behavior, attention, or memory. Children with ASD and epilepsy often exhibit various behavioral problems, including disruptive behaviors, tantrums, aggression, self-injury, hyperactivity, impulsivity, and non-compliance with rules [45]. Behaviors such as aggression, impulsivity, and non-compliance with rules increase their stress levels and have a negative impact on the quality of life of individuals with ASD and epilepsy [46]. Some studies have indicated that sleep disorders can worsen behavioral problems in individuals with ASD and epilepsy. For example, Fadini et al. [47], when examining the relationship between sleep and behavior in a sample of 45 children with ASD and epilepsy, concluded that sleep disorders were linked to thinking and behavioral problems. Similarly, Sikora et al. [48], in a sample of 1193 children with ASD, found that children with ASD and epilepsy who had a sleep disorder exhibited more internalizing and externalizing behavior problems, as well as poorer adaptive skill development compared to children with ASD and epilepsy without sleep problems. Park et al. [49] analyzed sleep disorders, their correlates, and psychopathological comorbidities in 166 children with ASD and epilepsy compared to 111 siblings without pathology. They found that children with sleep disorders were more likely to display aggressive behaviors, internalizing and externalizing problems, and behavior problems compared to those without sleep problems.

On the other hand, children with ASD and epilepsy who experience sleep disorders exhibit symptoms of hyperactivity, inattention, and memory problems, with around 30% of them reporting cognitive issues encompassing one or more of these functions [49,50]. Various studies have found that children with ASD and epilepsy experience a high degree of sleep problems, including variable sleep patterns, delayed sleep onset, insomnia, and, consequently, daytime sleepiness [50,51,52]. Consistent with these findings, it has been noted that epilepsy symptoms can exacerbate sleep disorders in individuals with ASD. For example, a high degree of hyperactivity could delay sleep onset and, over time, lead to insomnia [52]. Therefore, the presence of sleep disorders at an early age negatively influences the development of attention and memory processes. DeVincent et al. [53], when exploring the relationship between sleep problems and cognitive symptoms, found that children with ASD, epilepsy, and sleep difficulties also had higher rates of hyperactivity and cognitive dysfunction compared to children without sleep problems. Similarly, Goldman et al. [54], when assessing the potential effect of sleep disorders in a cohort of children with ASD and epilepsy, identified that those with disrupted sleep patterns exhibited more inattention, hyperactivity, and restricted/repetitive behaviors. In the following table (Table 2), sleep disorders, their affected neurotransmitters, behavioral characteristics, and their treatment are reflected.

These findings support the notion that improving sleep in the pediatric population aims to reduce behavioral problems, which, in turn, helps alleviate stress in individuals with ASD and epilepsy and enhances family functioning. A recent report proposing a practical approach to the identification, assessment, and management of insomnia in children and adolescents with ASD suggests that pharmacological therapy (such as melatonin) may be recommended in certain cases, especially in those individuals who experience epileptic seizures during sleep phases [50]. Behavioral interventions, such as sleep hygiene, have been shown to be effective in addressing sleep difficulties in children with ASD and epilepsy [51]. In this regard, parents of children with ASD, epilepsy, and sleep disorders can benefit from tools to manage these challenges. Treatment approaches for sleep disorders in the population with both ASD and epilepsy often involve a combination of behavioral interventions, environmental adjustments, and, in some cases, pharmacological options. Behavioral interventions encompass strategies like establishing consistent bedtime routines, improving sleep hygiene, and utilizing relaxation techniques. Environmental adjustments may include creating a comfortable and sensory-friendly sleep environment. In certain instances, pharmacological therapies, such as melatonin supplementation, may be considered, especially for individuals prone to seizures during sleep phases. The choice of treatment depends on the specific sleep disorder, its underlying causes, and individual needs, underscoring the importance of a personalized and multidisciplinary approach to address the complex interplay of these conditions.

## 4. Discussion

The primary objective of this systematic review was to analyze the available evidence regarding the relationship between sleep disorders and neurobiological and cognitive alterations in the pediatric population with ASD. Additionally, the specific objectives were to examine the neurobiological alterations that occur due to sleep disorders and to describe the dysfunctions in cognitive processes in children diagnosed with ASD and epilepsy.

Regarding the neurobiological alterations present in individuals with these comorbid disorders, scientific literature has demonstrated that, in pediatric neurological conditions, the impact of sleep disorders involves dysregulation of a significant portion of the nervous system. This, in turn, signifies dysfunction in the GABAergic and serotonergic systems, implying a disruption of the hormonal system, specifically the one responsible for melatonin regulation [55,56,57]. However, research in this field is very limited due, in part, to the scarcity of studies, the small number of homogeneous samples, and the variability in the manifestations of neurological alterations in clinical variables and their etiology, making the studies inconclusive [58,59]. Nevertheless, this dysregulation of different neurological systems negatively impacts the developmental maturation of individuals with ASD and epilepsy, as it affects the learning processes [60], causing the symptoms of ASD itself to be more pronounced with greater disruptions in communication and language or in social behavior [61].

Regarding the cognitive changes due to sleep disorders (SD), it is noteworthy that they are part of the current definition of this comorbidity and have a significant impact on children with ASD and epilepsy adaptation to their environment, their relationships with peers, and cognitive development. In the past decade, the negative impact of early epilepsy on cognitive capacity has been confirmed, establishing it as a predictor of difficulties in the development of these functions in children with ASD and epilepsy [62]. All of this results in cognitive processes such as attention or memory showing reduced performance compared to the ASD population without epilepsy. This may be because, in childhood, neuronal plasticity mechanisms are at their peak, trying to consolidate attention and memory mechanisms via the assimilation of environmental stimuli [63]. However, the existence of an SD will have a negative effect and reduce the ability to integrate knowledge from acquired context stimuli, as it will reduce cognitive alertness, preventing proper attentional focus, limiting the information captured by the child’s sensory system from being recorded and stored in memory [64].

The strengths and limitations of this study should be considered. A limitation of this study is that it relied on a review of existing literature, so the availability and quality of the included studies may have influenced the results. Since no original research was conducted and no new data were collected, there is a possibility that some relevant studies were omitted or that the results are influenced by publication bias. Additionally, the quality and methodology of the studies included in the review may vary, which could affect the reliability of the findings. Therefore, the results should be interpreted with caution and take into account the inherent limitations of the literature review. However, a strength of this study is that it considered recent and relevant research, providing an updated insight into the neurobiological causes of sleep disorders, cognitive impairments, and emotional disturbances in families with children with ASD and epilepsy. By including previous high-quality studies, a solid foundation was established for understanding the phenomena studied, and a broader coverage of the existing literature was achieved. Furthermore, by reviewing both international and national studies, a global perspective of research in the field was obtained, increasing the generalizability of the results. Another strength of this study is that it addressed multiple aspects related to sleep disorders, cognitive impairments, and emotional disturbances in the studied population. By examining the neurobiological causes and potential relationships between these phenomena, a more comprehensive and holistic understanding of the issue was achieved. This allows for the identification of key areas for intervention and the development of more effective therapeutic strategies.

The results of this study have important implications both in the clinical and research domains. In the clinical realm, it highlights the importance of assessing and addressing sleep disorders and neurological and cognitive impairments in individuals with ASD and epilepsy. Healthcare professionals should consider these areas and adopt a comprehensive approach to improve the quality of life for affected individuals and their families. Regarding future research, there is a suggestion to delve deeper into the understanding of the underlying neurobiological causes of sleep disorders in this population. It is necessary to review the action of neurotransmitters such as serotonin, GABA, and other substances like dopamine, which play a crucial role in sleep behavior disorders. In addition, studies exploring specific therapeutic interventions aimed at improving the quality of sleep patterns in individuals with ASD and epilepsy are needed. Furthermore, protocols for assessing cognitive processes affected by sleep disorders, such as memory, attention, or executive functions, should be established. Longitudinal studies could also be conducted to better comprehend the evolution of sleep disorders and cognitive impairments over time in individuals with ASD and epilepsy, comparing cognitive impairments and neurobiological activity based on types of sleep disorders. These investigations would allow for the assessment of the long-term effectiveness of different intervention approaches and provide insights into how these phenomena develop and change over time.

In conclusion, this review underscores the importance of understanding the neurobiological causes of sleep disorders and neurobiological and cognitive impairments in individuals with ASD and epilepsy. The results support the existence of relationships between these phenomena and emphasize the need for a comprehensive approach to the assessment and treatment of these conditions. Further research is required to deepen our understanding and develop more effective interventions that comprehensively address sleep disorders and neurobiological and cognitive impairments in individuals with ASD and epilepsy.

## Figures and Tables

**Table 1:** Summary of selected articles.

Authors	Year	N	Age (Years)	Objective	Abstract	Journal	Impact Factor (2022)
**Neurobiological alterations of Sleep Disorders in children with ASD and epilepsy**							
Saravanapandian et al. [33]	2021	27	6	To analyze abnormal sleep physiology in children with ASD and other related disorders.	Abnormal sleep physiology in children with ASD was investigated, and altered patterns of sleep and wakefulness were found.	Molecular autism	6.8
McCauley et al. [34]	2004	123	8	To map the linkage imbalance in the GABA(A) subunit cluster of the 15q12 receptor of children with ASD and epilepsy and its association with sleep disorders	A map of linkage imbalance in the GABA(A) receptor 15q12 was performed in people with ASD and epilepsy and was related to sleep disorders.	American journal of medical genetics. Part B, Neuropsychiatric genetics	3.0
Chen et al. [35]	2017	133	12	Use blood serotonin as an endophenotype to identify rare variants involved in ASD and epilepsy.	Blood serotonin was used as an endophenotype to identify genetic variants associated with ASD and epilepsy.	Molecular autism	6.8
Hranilović et al. [36]	2008	63	6	To examine hyperserotoninism in ASD and epilepsy and its relationship to serotonin-related gene variants.	Hyperserotoninism in ASD is comorbid with epilepsy, and its relationship with serotonin-related gene variants was investigated.	Collegium antropologicum	N/D
Prasad et al. [37]	2009	5	5	To study the enhanced activity of human serotonin transporter variants associated with ASD.	The enhanced activity of human serotonin transporter variants associated with ASD was studied.	Philosophical Transactions of the Royal Society of London. Series B, Biological sciences	6.0
Verma et al. [38]	2014	422	4–17	To investigate the dimorphic sexual effect on the genetic association of monoamine oxidase A (MAOA) markers with ASD and epilepsy.	We investigated the dimorphic sexual effect on the genetic association of MAOA markers with ASD and epilepsy.	Progress in neuro-psychopharmacology and biological psychiatry	4.2
Goldman et al. [39]	2014	24	7	Characterize melatonin in children with ASD in relation to sleep.	Melatonin was characterized in children with ASD in relation to sleep, finding differences in endogenous production and pharmacokinetics.	Journal of autism and developmental disorders	3.8
Goldman et al. [40]	2017	28	13	Characterize sleep in children with ASD.	Sleep was characterized in children with ASD, observing differences in sleep duration and quality.	Journal of autism and developmental disorders	3.8
Braam et al. [41]	2018	60	5–17	To investigate the low level of maternal melatonin and its relationship with the risk of epilepsy and ASD in children.	Low maternal melatonin levels and their relationship to the risk of ASD and epilepsy in children were investigated.	Research in developmental disabilities	2.2
Jonsson et al. [42]	2014	1771	7	Acetylserotonin O-methyltransferase (ASMT) associated with ASD traits in children from a Swedish cohort.	An association between ASMT and ASD traits was found in children from a Swedish cohort.	Psychiatric genetics	2.2
Veatch et al. [43]	2015	15	3–15	To examine genetic variation in melatonin pathway enzymes in children with ASD and epilepsy and delayed sleep onset.	We examined genetic variation in melatonin pathway enzymes in children with ASD and epilepsy and delayed sleep onset.	Journal of autism and developmental disorders	3.8
Gunes et al. [44]	2019	112	2–18	To assess sleep problems in children with ASD and their correlation with attention deficit hyperactivity disorder.	Sleep problems in children with ASD and their correlation with ADHD were evaluated, finding differences in sleep patterns.	Neuropsychiatric disease and treatment	3.2
**Cognitive alterations of Sleep Disorders in children with ASD and epilepsy**							
Qin et al. [45]	2022	54	2–6	Analyze brain networks in preschool children with ASD.	Brain networks were investigated in preschool children with ASD, and differences in functional connectivity were found.	Frontiers in psychiatry	3.5
Miner et al. [46]	2023	24	6–12	To evaluate the relationship between sleep disorders and the severity and common behaviors in ASD.	The relationship between sleep disorders and the severity of behaviors in ASD was evaluated, finding associations between sleep patterns and behaviors.	Research square	2.3
Fadini et al. [47]	2015	101	4–18	Investigate the influence of sleep disorders on the behavior of individuals with ASD.	The influence of sleep disorders on the behavior of individuals with ASD was investigated, finding correlations between sleep disorders and behavior.	Frontiers in Human Neuroscience	3.6
Sikora et al. [48]	2012	3452	4–10	To examine the relationship between sleep problems and daytime behavior in children of different ages with ASD.	The relationship between sleep problems and daytime behavior in children with ASD of different ages was examined, finding correlations between both.	Pediatrics	6.6
Park et al. [49]	2012	166	4–15	To investigate sleep problems and their relationship with comorbid psychopathology in children with ASD.	Sleep problems and their relationship with psychopathology in children with ASD were investigated, finding associations between sleep problems and comorbidity.	Research in Autism Spectrum Disorders	2.4
da Silveira Cruz-Machado et al. [50]	2021	40	6–8	To study disrupted nocturnal melatonin in ASD and its relationship with tumor necrosis factor and sleep disorders.	Disrupted nocturnal melatonin was studied in ASD and its relationship with tumor necrosis factor and sleep disorders.	Journal of Pineal Research	14.7
Bin Eid et al. [51]	2022	29	6–16	To analyze the alterations in cortisol profiles in mothers of children with ASD related to the poor quality of sleep in children.	Alterations in cortisol profiles in mothers of children with ASD related to the children’s sleep quality were analyzed.	Healthcare (Basel, Switzerland)	2.3
Page et al. [52]	2020	13	2.5	To investigate the relationship between non-rapid eye movement sleep and the risk of autism spectrum disorder in early development.	The relationship between non-rapid eye movement sleep and ASD risk in early development was investigated, finding differences in EEG topography.	Brain and behavior	2.5
DeVincent et al. [53]	2007	112	3–5	To evaluate sleep disorders and their relationship with psychiatric symptoms in preschoolers with ASD and community controls.	Sleep disorders and their relationship with psychiatric symptoms were evaluated in preschoolers with ASD and controls, finding associations.	Journal of Child Neurology	2.0
Goldman et al. [54]	2009	58	4–10	Define the sleep phenotype in children with ASD.	The sleep phenotype in children with ASD was defined, highlighting differences in sleep latency and nocturnal disturbances.	Developmental neuropsychology	2.0

**Table 2:** Characteristics related to sleep disorders in the pediatric population with ASD and epilepsy.

Sleep Disorder	Affected Neurotransmitters	Characteristic Behavior	Common Treatment
Insomnia	Serotonin	Difficulty falling asleep, frequent awakenings during the night.	Cognitive-Behavioral Therapy for Insomnia (CBT-I), medications under medical supervision.
Nightmares	Norepinephrine	Sudden awakenings with fear or anxiety.	Nightmare Desensitization Therapy, cognitive-behavioral therapy.
Night Terrors	GABA	Loud screams or extreme agitation during sleep without fully waking up.	Education about the disorder, sleep environment adjustments, and cognitive-behavioral therapy.
Sleep Apnea	N/A	Loud snoring, breathing pauses during sleep.	CPAP (Continuous Positive Airway Pressure), lifestyle changes.
